# Probing the Production of Amidated Peptides following Genetic and Dietary Copper Manipulations

**DOI:** 10.1371/journal.pone.0028679

**Published:** 2011-12-16

**Authors:** Ping Yin, Danielle Bousquet-Moore, Suresh P. Annangudi, Bruce R. Southey, Richard E. Mains, Betty A. Eipper, Jonathan V. Sweedler

**Affiliations:** 1 Department of Chemistry, Beckman Institute, University of Illinois at Urbana-Champaign, Urbana, Illinois, United States of America; 2 Department of Neuroscience, University of Connecticut Health Center, Farmington, Connecticut, United States of America; University of Leuven, Rega Institute, Belgium

## Abstract

Amidated neuropeptides play essential roles throughout the nervous and endocrine systems. Mice lacking peptidylglycine α-amidating monooxygenase (PAM), the only enzyme capable of producing amidated peptides, are not viable. In the amidation reaction, the reactant (glycine-extended peptide) is converted into a reaction intermediate (hydroxyglycine-extended peptide) by the copper-dependent peptidylglycine-α-hydroxylating monooxygenase (PHM) domain of PAM. The hydroxyglycine-extended peptide is then converted into amidated product by the peptidyl-α-hydroxyglycine α-amidating lyase (PAL) domain of PAM. PHM and PAL are stitched together in vertebrates, but separated in some invertebrates such as *Drosophila* and *Hydra*. In addition to its luminal catalytic domains, PAM includes a cytosolic domain that can enter the nucleus following release from the membrane by γ-secretase. In this work, several glycine- and hydroxyglycine-extended peptides as well as amidated peptides were qualitatively and quantitatively assessed from pituitaries of wild-type mice and mice with a single copy of the *Pam* gene (PAM^+/−^) via liquid chromatography-mass spectrometry-based methods. We provide the first evidence for the presence of a peptidyl-α-hydroxyglycine *in vivo*, indicating that the reaction intermediate becomes free and is not handed directly from PHM to PAL in vertebrates. Wild-type mice fed a copper deficient diet and PAM^+/−^ mice exhibit similar behavioral deficits. While glycine-extended reaction intermediates accumulated in the PAM^+/−^ mice and reflected dietary copper availability, amidated products were far more prevalent under the conditions examined, suggesting that the behavioral deficits observed do not simply reflect a lack of amidated peptides.

## Introduction

Neuropeptide production typically involves endoproteolytic cleavage of prohormones by prohormone convertases, followed by exoproteolytic cleavage by carboxypeptidase E. As the prohormone and its cleaved products traverse the secretory pathway, additional post-translational modifications occur. One common modification is C-terminal α-amidation, a reaction catalyzed only by peptidylglycine α-amidating monooxygenase (PAM) [1], [2]. The C-terminal amide group often increases the affinity of the peptide for its receptor, extends its half-life, and is essential for biological activity [3]. Eliminating the PAM gene prevents amidated peptide synthesis and is lethal in *Drosophila* [4] and mouse [1]. PAM heterozygous (PAM^+/−^) mice, with half the PAM activity of wild-type (WT) mice, survive to adulthood and reproduce [3]. However, PAM^+/−^ mice are unable to maintain body temperature in a cold room and show increased anxiety-like behavior [5], [6]. In vertebrates, PAM is a Type I integral membrane protein; its luminal catalytic domains and unstructured cytosolic domain are highly conserved. A γ-secretase-mediated cleavage within the PAM transmembrane domain generates a cytosolic fragment that accumulates in the nucleus and is thought to affect gene expression [5], [7], [8], [9].

PAM has two enzymatic domains, peptidylglycine-α-hydroxylating monooxygenase (PHM) and peptidyl-α-hydroxyglycine α-amidating lyase (PAL). PHM converts glycine-extended peptides into hydroxyglycine-extended peptides by using molecular oxygen to hydroxylate the α-carbon of the C-terminal glycine in a copper- and ascorbate-dependent reaction. The hydroxyglycine-extended peptide is then converted into the corresponding amidated peptide and glyoxylate by PAL [10], [11]. Although several glycine-extended peptides (e.g., TRH-Gly [5], [12], [13] and gastrin-Gly [14], [15]) have been detected using radioimmunoassays, they have not been studied systematically. Hydroxyglycine-extended peptides have not been detected *in vivo*, suggesting that the product of the monooxygenase reaction might be passed directly to the lyase. In addition, PAM is amongst the small number of enzymes that require copper for their catalytic function [16], [17], [18], [19], and behavioral deficits similar to those observed in PAM^+/−^ mice were observed in WT mice kept on a copper-deficient diet [5]. Whether the deficits observed in PAM^+/−^ and copper-deficient mice reflect altered levels of amidation or changes in the regulatory processes responsive to PAM and copper is not yet clear.

Liquid chromatography (LC)-mass spectrometry (MS) provides both qualitative and quantitative information and enables sensitive and accurate determination of neuropeptide forms [20], [21], [22], [23], [24]. Here we used two LC-MS based neuropeptidomics approaches to evaluate the effects of PAM haploinsufficiency and dietary copper deficiency on the levels of several glycine- and hydroxyglycine-extended peptides as well as amidated peptides in the mouse pituitary. We applied a standard isotope labeling approach with LC-MS analysis to perform relative quantitation for amidated peptides in PAM^+/−^ mice maintained on a normal diet compared to WT mice. We also found that endogenous intermediates were present at much lower levels than their amidated counterparts, with the large dynamic range of peptide concentrations making their identification and quantitation challenging. In this case, following LC separation, we employed both matrix-assisted laser desorption/ionization (MALDI) time-of-flight (TOF) MS and electrospray ionization (ESI) ion-trap (IT) MS to identify the intermediate peptides. After identification, the levels of glycine- and hydroxyglycine-extended peptides were compared to those of amidated products using MS-based peak intensities, and differences were correlated to genetic and/or dietary manipulations. The standard labeling approach requires multiple sample processing steps and is well suited for assays of peptides at higher and similar concentrations; however, these additional steps can cause peptides already at low levels to be reduced to a point below the MS detection limit [20], [25]. Therefore, we used a label-free approach to provide relative levels of the partially processed peptides [26], [27]. We identified hydroxyglycine intermediates in pituitary lysates, arguing against their transfer directly from PHM to PAL. Our results demonstrate that glycine-extended peptides accumulate when PAM levels are low and/or when copper availability is limited but that amidated products still predominate.

## Materials and Methods

### Ethics Statement

This study was carried out in strict accordance with the recommendations in the Guide for the Care and Use of Laboratory Animals of the National Institutes of Health. The Animal Protocols used for this study, numbers 2006-229 and 2009-517, both entitled Biochemistry and Physiology of Peptide Amidation, conformed to the University of Connecticut Health Center Animal Care Committee guidelines and the ARRIVE guidelines have been followed.

### Materials

HPLC grade solvents such as water and acetonitrile (ACN) with 0.1% formic acid (FA) and 0.01% trifluoroacetic acid (TFA) were purchased from Fisher Scientific (Pittsburgh, PA, USA). The MALDI matrix, α-cyano-4-hydroxycinnamic acid (α-CHCA), was from Sigma-Aldrich (St. Louis, MO, USA). Synthetic ACTH(1-13)-Gly [ACTH(1-14)] (AnaSpec, Fremont, CA, USA) was converted into ACTH(1-13)-Gly-OH using PHM catalytic core purified from stably transfected Chinese hamster ovary cell medium [3], [4], [28]. The 1.0 mL reaction mixture contained 1.0 µM CuSO_4_, 0.5 mM ascorbic acid, 100 µM peptide, 12 µg PHMccQ170A, and 0.09 mg/mL bovine liver catalase in 150 mM NaMES (Sigma-Aldrich), pH 5.0, and the reaction was allowed to proceed for 5 h at 37°C. The reaction was stopped by adding an equal volume of 0.1% TFA. The peptide was adsorbed to a SepPak C_18_ μBondapak cartridge (Waters Corporation, Milford, MA, USA), which was then washed with 0.1% TFA and eluted with 80% ACN in 0.1% TFA. Fractions containing peptide were pooled (based on A_280_) and lyophilized.

### Animals

Adult male PAM^+/−^ mice were used for all reported experiments. Male PAM^+/−^ mice (back-crossed more than 15 generations into C57BL/6J mice, Jackson Laboratory, Bar Harbor, ME, USA) were mated with WT female C57BL/6J mice (Jackson Laboratory); weanlings were genotyped as described [6]. Dietary copper manipulations were carried out as described [5], [6]. WT and PAM^+/−^ mice were made copper deficient by keeping them on a copper-deficient diet, Harlan Teklad TD80388 (Harland Laboratories, Inc., Frederick, MD, USA), with reverse osmosis treated water for 9–10 wk. Copper-supplemented mice were provided with Harlan Teklad control diet #2018 or normal mouse chow and deionized reverse osmosis treated water supplemented with 300 ppm CuSO_4_•5H_2_0 (70 ppm Cu) for 14–16 d. Control mice were provided with normal mouse chow or Harlan Teklad control diet #2018 and deionized reverse osmosis treated water [6]. Mice were group housed, with lights off from 7 pm to 7 am, room temperature at 20–22°C, and were weighed so that control and test groups were of equal average weight. They were brought to the laboratory in their home cages and allowed to acclimate for several hours to avoid any effect of anesthetics and circadian rhythms on the pituitary. Mice were sacrificed by decapitation between 10 am and 2 pm.

### Sample Preparation

According to genotype and diet, pituitaries were harvested, pooled into a group containing 2–4, and homogenized into 100 µL/pituitary freshly made ice-cold acidified acetone (acetone∶H_2_O∶HCl = 40∶6∶1, v/v/v) using a ground glass homogenizer and pestle. The homogenizer was rinsed with an additional 100 µL/pituitary of the same solution. Following centrifugation at 1,000×g for 90 min, supernatants were transferred to a new glass tube and subjected to vacuum centrifugation for 90 min to remove the acetone; the remaining sample was frozen and lyophilized overnight. For analysis, the lyophilized samples were dissolved into 2% ACN aqueous solution with 0.1% FA and 0.01% TFA (20 µL/pituitary). For label-free quantitation, eight separate sets of WT and PAM^+/−^ mice kept on a normal diet were analyzed; three separate sets of PAM^+/−^ mice kept on a normal or copper-supplemented diet were compared; and three separate sets of WT and PAM^+/−^ mice kept on a normal or copper-deficient diet were compared, with the number of biological replicates defined for each experiment below. Each sample in the comparison set included the same number of pituitaries (2–4 pituitaries/sample as mentioned above) from mice of different genotypes or diets but raised during the same time.

For labeling-based quantitation of amidated peptides, samples were grouped based on the isotopic chemical labels they would receive. For these experiments, only genotype effects were investigated as the labeling reaction was performed to compare the peptide levels in PAM^+/−^ mice with those in WT mice on a normal diet as described previously [23]. The sample solution was adjusted to pH 9 with 1 M phosphate buffer and 1 M NaOH. The control (e.g., WT on a normal diet) and the experimental (e.g., PAM^+/−^ on a normal diet) samples were labeled with succinic anhydride (SA)—5 µL of 2 M light form (SA-H) and heavy form (SA-D)—in dimethyl sulfoxide (DMSO), respectively. The reagents were thoroughly mixed by vortex and centrifuged, followed by incubation for 10 min at 20–22°C. After incubation, the addition of NaOH for adjusting the sample solutions to pH 9 and SA for labeling was repeated four times. The remaining SA was quenched by 20 µL of 2.5 M glycine for 40 min at 20–22°C. The labeled control and experimental samples were then combined, followed by adjustment to pH 9. Next, 10 µL of 2 M hydroxylamine solution was added before the mixture was reacted at 20–22°C for 30 min. Following all of the reactions, the combined sample was desalted and pre-concentrated by using a PepClean C18 spin column (Pierce, Rockford, IL, USA). The solvent of the eluent from the spin column was evaporated in a SpeedVac (Thermo Fisher Scientific, Waltham, MA, USA) and the labeled peptides were reconstituted into 2% ACN aqueous solution with 0.1% FA and 0.01% TFA.

### LC-MS Analysis of Amidated Peptides

After labeling the samples were analyzed with an Ultraflex II MALDI-TOF/TOF mass spectrometer (Bruker Daltonics, Billerica, MA) in the mass range of 600–5000 Da. Specifically, 1 µL of each sample mixed with 1 µL of 10 mg/mL α-CHCA in 70% ACN aqueous solution containing 0.1% FA and 0.01% TFA was spotted onto a gold MALDI target. Each sample was spotted onto three MALDI sample spots to provide technical replicates. The spectra obtained with a total of 500 laser shots were summed. As the neuropeptides in mouse pituitary have been well characterized, peptides were identified by matching their parent ion masses before labeling within 20 ppm of the theoretical masses of the mouse pituitary peptides previously reported [29], [30]. SA-based isotopic labeling introduced a mass difference of 4 Da between the light- and heavy-labeled samples in MALDI-TOF MS analysis. For each peptide, quantitation was performed by calculating the peak intensity ratio of the light over heavy forms. Eight biological replicates were used to study the peptides in WT versus PAM^+/−^ mice on a normal diet. Statistical tests with the technical and biological replicates were performed as previously described [23]. Briefly, a two-stage mixed model commonly used in microarray analysis was applied here. In the first stage, the general differences between MS runs composed of labeling and treatment combinations were removed. The second stage involved a mixed model that was fitted to each peptide in terms of genotype.

### LC-MS Analysis of Glycine-extended and Hydroxyglycine-extended Peptides

Endogenous glycine-extended peptides were identified by signature fragment ion mass matching and elution order, as well as the widely used tandem MS (MS^2^) matching approach. For LC ESI-IT MS and MS^2^, analyses were performed using a capillary LC system (CapLC, Micromass, Manchester, UK) coupled to an HCTultra mass spectrometer (Bruker Daltonics). Endogenous hydroxyglycine-extended peptides were identified by parent ion mass matching, parent ion instability, and elution order with the CapLC coupled to the Ultraflex II MALDI-TOF/TOF mass spectrometer described above.

Peptide standards consisting of glycine- and hydroxyglycine-extended forms were used to assist the identification of partially processed peptides by providing information on signature fragment ions and elution order; here, 2 µL of 1 µM ACTH(1-13)-Gly and 2 µL of 10 µM ACTH(1-13)-Gly-OH were added to 6 µL of pituitary extract from WT mice. The mouse pituitary extracts or the spiked samples were separated by capillary LC with Solvent A (95% water, 5% ACN, 0.1% FA, and 0.01% TFA), and Solvent B (5% water, 95% ACN, 0.1% FA, and 0.01% TFA). The 45-min gradient was programed from 3% B to 20% B in 5 min, to 40% B in another 19 min, continued to 80% B in 7 min, and held at 80% B for 3 min before ramping back to 3% B. The fractions were immediately subjected to ESI-IT MS or MALDI-TOF/TOF MS, followed by MS^2^ analyses. The fragment ion mass matches were considered positive when within 0.2 Da from the ESI-IT MS^2^ analysis, and the parent ion mass matches when within 5 ppm from the MALDI-TOF MS analysis.

To test the stability of hydroxyglycine-extended peptide, 0.5 µL of 100 µM ACTH(1-13)-Gly-OH aqueous solution with 0.1% FA and 0.01% TFA (pH∼3) was deposited on a steel MALDI plate with 0.5 µL of 10 mg/mL α-CHCA in ACN aqueous solution (ACN/H_2_O = 70/30, v/v). The air-dried sample spot was analyzed by MALDI-TOF MS in the linear and reflectron modes, sequentially. The laser power was consistent during the acquisition, and 500 laser shots were summed for each spectrum. In addition, 50 µL of 100 µM ACTH(1-13)-Gly-OH in acidic aqueous solution was mixed with 50 µL of ACN for ESI-IT MS analysis with direct infusion. The mass-to-charge ratios (*m/z*) of doubly charged ACTH(1-13)-Gly-OH and its amidated form, ACTH(1-13)-NH_2_, were used as parent ions to be first isolated and then fragmented. The most intense fragment ion in this first-step fragmentation was again isolated and then further fragmented.

The identified peptides were quantified by a label-free MS-based approach using LC-MALDI-TOF MS [26], [27]. Briefly, 5 µL samples prepared from the pituitaries of mice of different genotypes or mice maintained on different diets were separated using the CapLC system described above. A total of 24 1-min fractions, collected between 20 and 45 min, were spotted for MALDI-TOF MS analysis. The α-CHCA (1 µL of 10 mg/mL) was added to each air-dried sample spot. The AutoeXecute function in the Ultraflex II mass spectrometer was used to automatically acquire MS data for each sample in the mass range of 900–2500 Da. To obtain reproducible results for quantitative analysis, sets of pituitary extracts prepared from mice of different genotypes or mice kept on different dietary regimens were subjected to parallel analyses with identical parameters from sample handling to LC-MS analysis. The amidated peptides were first confirmed by MALDI-TOF MS^2^, and then used for internal calibration. The peak annotations to glycine- or hydroxyglycine-extended peptides were made by parent ion mass matches within 5 ppm and via peak elution order. Intensities of peaks annotated to one peptide but eluted in several consecutive fractions were summed after normalization. The relative amounts of partially processed peptides compared to their amidated forms were indicated by the ratio of their corresponding intensities (glycine-extended peptide/amidated peptide or hydroxyglycine-extended peptide/amidated peptide) within the same sample.

To minimize the effects of experimental error, the comparison of glycine-extended peptide relative levels under different manipulations was conducted within pairs of samples that were prepared and analyzed at the same time. For example, a ratio for WT to PAM^+/−^ mice on a normal diet was calculated by dividing the ratio of the glycine-extended peptide peak to the amidated peptide peak for WT by the same ratio for PAM^+/−^. Statistical tests were then performed with this second ratio across biological replicate pairs analyzed at different times. Because the ratio of the glycine-extended peptide to the amidated peptide for WT was almost zero in most cases, we divided the ratio for WT by the ratio for PAM^+/−^ in order to avoid infinite values during the statistical tests.

As with other peptides, the abundance of partially processed joining peptide (JP) peptide was analyzed as a ratio to the abundance of amidated peptide to minimize experimental error associated with different runs. In this case, the abundance ratio was analyzed with a left-censored log-normal regression model using SAS software (SAS Institute Inc., Cary, NC). The data was considered left-censored because peptides were only detected when the abundance surpassed the MS detection limit. Consequently, observations where the abundance of partially processed peptide present was less than the MS detection limit, and the amidated peptide was measured, were treated as left-censored. This model included the effects of partially processed peptides, genotypes, and interactions between partially processed peptide and genotype.

## Results

### Identification of Glycine-extended Peptides

**Figure 1:**
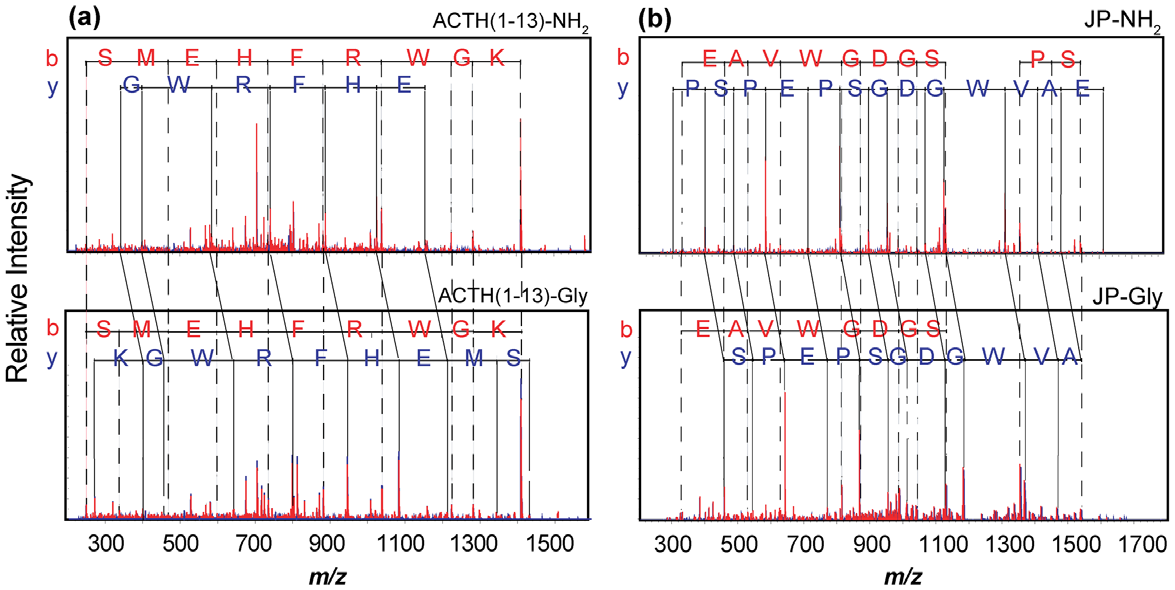
Analysis of mouse pituitary extracts using capillary LC coupled to ESI-IT MS^2^. Mass spectra of amidated (top) and glycine-extended (bottom) forms of (**A**) synthetic adrenocorticotropic hormone (ACTH) (1-13), and (**B**) endogenous joining peptide (JP). The assignment of b- and y-ions matches expected fragments within 0.2 Da.

The production of each amidated peptide must proceed through glycine- and hydroxyglycine-extended intermediates. Since levels of these intermediates were expected to be low, we utilized the pituitary, a tissue rich in peptides, for our studies; although the neuropeptides in mouse pituitary have been well characterized, these reaction intermediates have not been detected [28], [31], [32]. Characterization of these amidation intermediates is challenging, not only because of their low levels compared to the fully processed peptides, but also due to the restrictions imposed by MS detection limits and dynamic range. Nonetheless, among several observed amidation intermediates, glycine-extended joining peptide (JP-Gly) was identified with good sequence coverage via ESI-IT MS^2^ (Fig. 1).

For peptides that did not produce full coverage of their fragment ions with MS^2^ analysis, identifications were based on their parent ions, signature fragment ions, and elution order relative to the amidated forms. We determined this for amidated, glycine-extended, and hydroxyglycine-extended synthetic ACTH(1-13) before using this information for endogenous peptide identification. The signature fragment ions for glycine-extended peptides reflect the presence of a C-terminal glycine instead of a C-terminal amide. Therefore, the glycine-extended peptide is expected to consist of the same set of b ions, and a set of singly charged y ions, with a mass difference of ∼58 Da (C_2_H_5_NO_2_ minus NH_3_) as compared to its amidated product. These predictions were well supported by the fragment ions generated from synthetic ACTH(1-13)-Gly versus ACTH(1-13)-NH_2_, and from endogenous JP-Gly versus JP-NH_2_ (Fig. 1).

**Figure 2:**
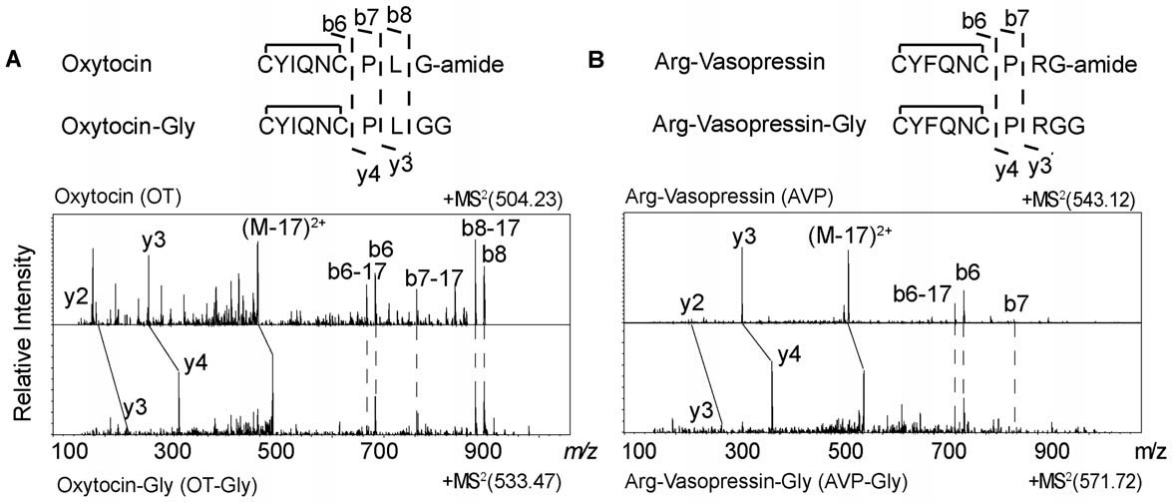
Analysis of mouse pituitary extracts using capillary LC coupled to ESI-IT MS^2^. Mass spectra of amidated (top) and glycine-extended (bottom) forms of (**A**) oxytocin and (**B**) arginine-vasopressin. The labeled b- and y-ions are the signature fragment ions used for their identification.

Based on the signature fragment ion assignments, we identified the endogenous glycine-extended forms of oxytocin (OT-Gly) and arginine-vasopressin (AVP-Gly) with LC-ESI-IT MS^2^. As shown in Figure 2A, identical b6, b7, and b8 ions between OT-Gly and OT-NH_2_, together with the singly charged y ions and doubly charged (M-17) ions of OT-Gly, with 58 and 29 Da mass differences, respectively, from those of OT-NH_2_, suggested the assignment of OT-Gly. The disulfide bond between cysteine 1 and cysteine 6 hindered cleavages to form smaller b ions and larger y ions. In addition, the identities of these glycine-extended peptides were confirmed by their LC elution times in comparison to those of synthetic peptides. ACTH(1-13)-NH_2_, ACTH(1-13)-Gly-OH, and ACTH(1-13)-Gly showed increasingly longer retention times on the reversed-phase column. Accordingly, we observed that JP-Gly, AVP-Gly, and OT-Gly eluted after the corresponding amidated peptides.

### Relative Quantification of Glycine-extended versus Amidated Peptides

**Figure 3:**
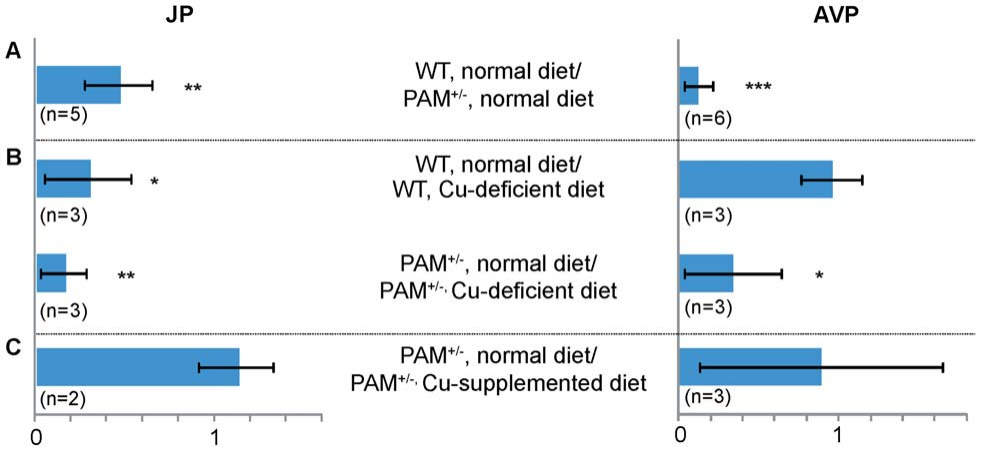
Effects of PAM haploinsufficiency and dietary copper (Cu) on the amidation of joining peptide (JP) and arginine-vasopressin (AVP). Pituitary samples were taken from WT and PAM^+/−^ mice maintained on normal, copper-deficient or copper-supplemented diets. In order to take into account differences in the sample preparation and mass spectrometry, for each peptide in each sample, the ratio of glycine-extended to amidated peptide was calculated, with higher numbers indicating an accumulation of glycine-extended peptide. The effects of (**A**) haploinsufficiency, (**B**) copper deficiency and (**C**) copper supplementation were then assessed by comparing the ratios between the pairs of samples to indicate the extent of the glycine-extended peptide accumulation. For example, (A) reports the WT normal diet value divided by the PAM^+/−^ normal diet value. N is the number of biological replicate pairs. Error bar, SEM. Student's *t*-test: ***, *p*<0.0005; **, *p*<0.05; *, *p*<0.1.

There are several approaches for comparing the relative levels of peptides, including stable isotope labeling and label-free approaches [20], [23], [26], [27], [33]. We used a label-free LC-MALDI-TOF MS approach here because it requires less sample processing and is well suited to characterizing peptides present at levels near the MS detection limit. Although neither form of oxytocin was observed by LC-MALDI-TOF MS, this technique provided enough sensitivity and resolution for us to quantify JP-Gly and AVP-Gly, relative to their amidated forms. Automated MS acquisition provided similar peptide profiles for samples from different runs, which shows the replicate reproducibility and the comparable amount of most peptides. As expected, in WT mice kept on a normal diet, levels of JP-Gly and AVP-Gly were very low when compared to levels of JP-NH_2_ and AVP-NH_2_, respectively. For example, JP-Gly levels were 100–1000-fold below JP-NH_2_ levels, and AVP-Gly levels were about 1000-fold below AVP-NH_2_ levels. This same ratio was calculated for PAM^+/−^ mice. If the level of PAM in the corticotropes or in the hypothalamic neurons that produce AVP is limiting, one would expect to see an increase in the ratio of glycine-extended to amidated peptide in the PAM^+/−^ mice. The JP-Gly/JP-NH_2_ ratio for WT mice was significantly smaller (*p*<0.05) than the same ratio for PAM^+/−^ mice, and the effect of PAM haploinsufficiency on the AVP-Gly/AVP-NH_2_ ratio was more dramatic, with a greater than 5-fold drop in this ratio (*p*<0.0005) (Fig. 3A).

Since the catalytic activity of PAM is dependent on copper, we next asked whether the ratio of glycine-extended to amidated peptide was altered by copper deficiency in WT or PAM^+/−^ mice (Fig. 3B). If a lack of copper limits the ability of PAM to catalyze this reaction, one would expect the glycine-extended to amidated peptide ratio to be increased by copper deficiency. In PAM^+/−^ mice, this is what was seen for JP: JP-Gly accumulated when these mice were copper deficient (*p*<0.05). For AVP-Gly, a trend towards accumulation of the glycine-extended peptide was observed (*p*<0.1) in the PAM^+/−^ mice. For WT mice, a trend towards accumulation of JP-Gly was observed with copper deficiency (*p*<0.1), but there was no evidence for accumulation of AVP-Gly in copper deficiency. Copper supplementation did not reduce the accumulation of JP-Gly or AVP-Gly observed in PAM^+/−^ mice, although there is a large variation for AVP-Gly (Fig. 3C). Overall, both lower levels of PAM and a copper-deficient diet induced accumulation of JP-Gly, while AVP-Gly accumulation was increased only by a reduction in PAM levels. Amidated peptide remained the major product even when PAM^+/−^ mice were made copper deficient.

### Identification of the Hydroxyglycine-extended Intermediates

**Figure 4:**
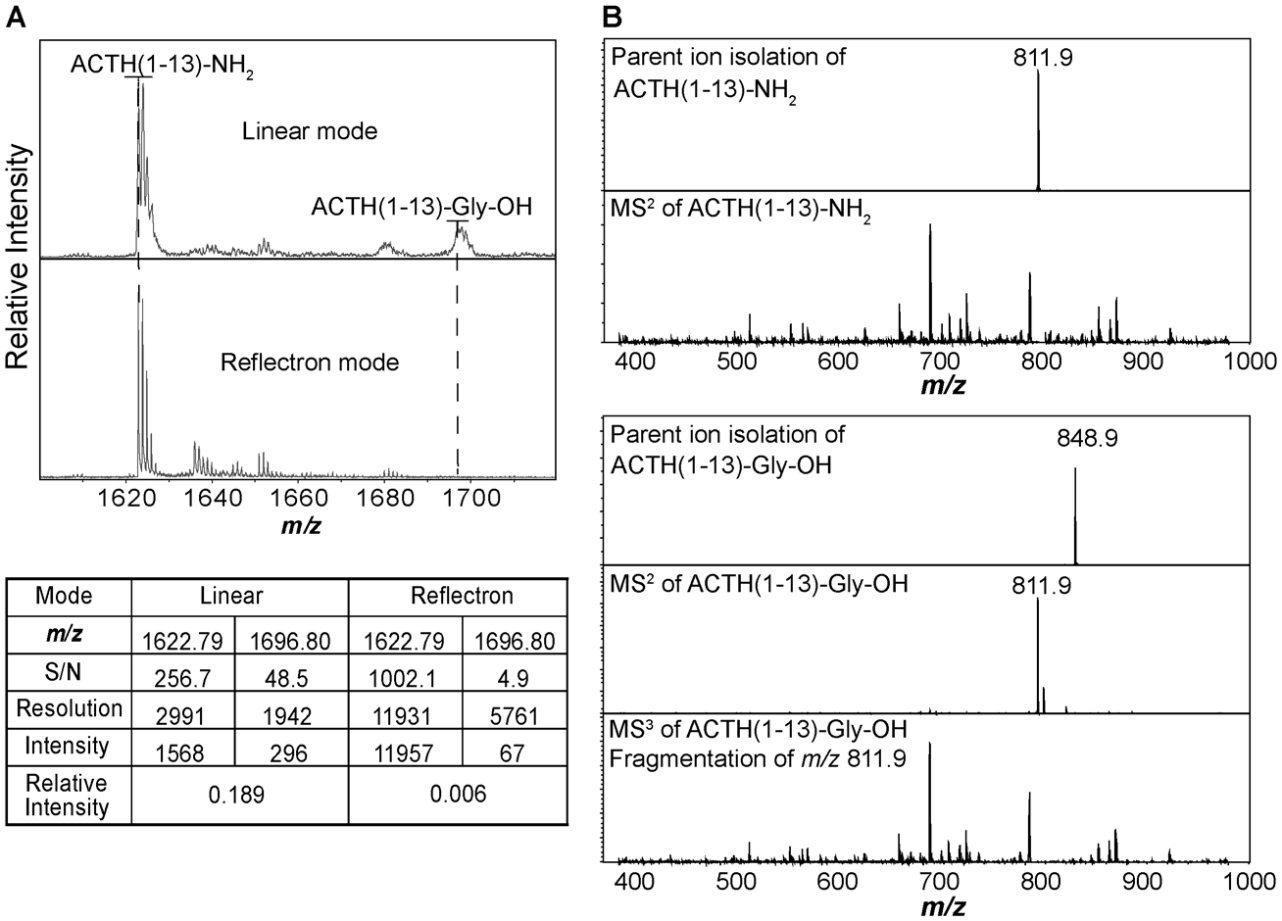
Conversion of ACTH(1-13)-Gly-OH to its amidated form ACTH(1-13)-NH_2_ during the measurement process when analyzed by MALDI-TOF MS and ESI-IT MS^2^. (**A**) The table shows the assessment of the amidated and hydroxyglycine-extended ACTH(1-13) peaks in MALDI-TOF MS. (**B**) With manual ESI-IT MS^2^, ACTH(1-13)-NH_2_ and ACTH(1-13)-Gly-OH were respectively isolated, and then fragmented. The MS^2^ of ACTH(1-13)-NH_2_ matches the MS^3^ of ACTH(1-13)-Gly-OH.

Although test tube studies and metabolic labeling revealed the two-step nature of the amidation reaction [28], hydroxyglycine-extended peptides have not been identified in tissue. Their presumed low abundance compared to their amidated forms makes measurement of hydroxyglycine-extended peptides challenging. In addition, it is known that hydroxyglycine-extended peptides are unstable in basic aqueous solutions, generating amidated peptide non-enzymatically [34]. Our data indicate that hydroxyglycine-extended intermediates are not stable in the gas phase during MS measurement and decompose to form amidated peptide. During MALDI-TOF MS analysis, ACTH(1-13)-Gly-OH produced from ACTH(1-13)-Gly using purified PHM was largely converted into ACTH(1-13)-NH_2_ (Fig. 4A).

We confirmed that ACTH(1-13)-Gly-OH decomposed during MS measurement by using the reflectron mode in our MALDI-TOF MS analysis. Just as with the γ-carboxyglutamate residue in a peptide [35], the ion lasts long enough to enter the field-free region and then fragments. In the linear mode, the peptide fragments reach the detector as a single peak with the hydroxyglycine-extended peptides. However, during reflectron operation, the decomposition products are separated well from the hydroxyglycine-extended peptides and dominate the MS detection. Therefore, we observed a greatly reduced relative intensity of ACTH(1-13)-Gly-OH compared to ACTH(1-13)-NH_2_ in the reflectron mode compared to the linear mode, which indicates that ACTH(1-13)-Gly-OH is converted into ACTH(1-13)-NH_2_ during MS analysis (Fig. 4A).

We further investigated the nature of this instability by using ESI-IT MS^2^, where we isolated the parent ions of either ACTH(1-13)-NH_2_ or ACTH(1-13)-Gly-OH, and then carried out corresponding MS^2^ and/or MS^3^ analysis. After conducting MS^2^ on the isolated ACTH(1-13)-Gly-OH parent ion, we observed a single peak corresponding to ACTH(1-13)-NH_2_. This peak produced the same fragment ions as the ones produced from MS^2^ of isolated ACTH(1-13)-NH_2_ (Fig. 4B). Both MALDI-TOF and ESI-IT observations support the idea that hydroxyglycine-extended peptides are detected with the corresponding amidated form in the same MS scan because of their decomposition. Using a similar criterion (a peak that decomposes into the amidated form), the expected elution order, and a parent ion mass match within 5 ppm by MALDI-TOF MS, we confirmed the identity of hydroxyglycine-extended JP (JP-Gly-OH) from mouse pituitary.

### Quantification of Hydroxyglycine-extended Precursors versus Amidated Peptides

**Figure 5:**
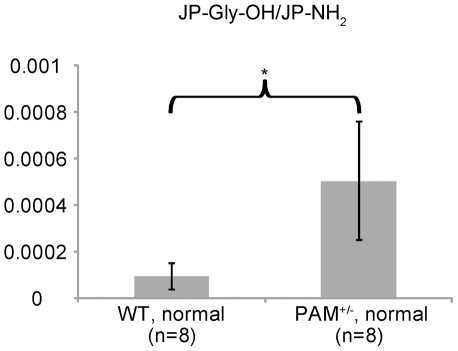
Expected abundance of JP-Gly-OH relative to JP-NH_2_ in the pituitaries of WT and PAM^+/−^mice on a normal diet. N is the number of biological replicates. Error bar, SEM. *, p<0.1.

In WT mice on a normal diet, JP-Gly-OH levels were generally at or below the MS detection limit, and were approximately 2000-fold (or more) below the levels of JP-NH_2_ (Fig. 5). When peptide levels were below the MS detection limit, an average ratio of hydroxyglycine-extended peptide to amidated peptide at the detection limit was used to allow all LC-MS runs to be included. In PAM^+/−^ mice, the levels of JP-Gly-OH were often above the detection limit and the ratio of JP-Gly-OH to JP-NH_2_ was 7.5-fold higher than the same ratio observed in WT mice (*p*<0.1) (Fig. 5A), suggesting that the amount of PAL had become limiting. In addition, levels of JP-Gly-OH were ∼70-fold lower than levels of JP-Gly (p<0.001), indicating that the monooxygenase reaction is a slower step in peptide amidation.

### Identification and Quantitation of Amidated Peptides under Different Manipulations

A total of 12 amidated peptides with or without other post-translational modifications, such as N-terminal acetylation, were identified in adult male mouse pituitary extracts with MS^2^ (Table 1). MALDI-TOF MS was then used to quantify the identified amidated peptides as a function of genotype. Compared to ESI, MALDI-TOF MS simplified the peptide profiles with only singly charged peptides [25], [36], [37], [38]. In this study, α-CHCA was used as matrix to provide homogenous distribution of peptides on the sample spots. The instrument parameters stayed the same throughout the analyses on all of the sample spots to ensure reproducible results. Only spectra with similar peptide profiles over a threshold of 1000 counts were summed to reflect the peptide relative levels. Although many reagents are available [23], [36], [39], SA was used for labeling quantitation in this study because it is stable and commercially available. Since the mass difference between the light and heavy forms of SA is about 4 Da, the singly labeled peptides in the control and experimental samples appear as a pair of peaks separated by 4 Da in the mass spectra (data not shown). Most of the observed peptides did not show significant changes between PAM^+/−^ and WT mice, except for three: alpha-melanocyte stimulating hormone (α-MSH), JP, and diAc-α-MSH, which were 1.42-, 1.56-, and 1.74-fold higher (*p*<0.05) in PAM^+/−^ mice compared to WT mice, respectively.

**Table 1:** Amidated peptides identified in adult male mouse pituitary extracts.

Precursor	Peptide name	Sequence	MH^+^_theor_.
Prooxytocin	Oxytocin	CYIQNCPLG-amide	1007.44
Provasopressin	Vasopressin	CYFQNCPRG-amide	1084.45
Proopiomelanocortin	DesAc-α-MSH	SYSMEHFRWGKPV-amide	1622.79
Proopiomelanocortin	Dehydro-α-MSH	Ac-dehydroS-YSMEHFRWGKPV-amide	1646.79
Proopiomelanocortin	α-MSH	Ac-SYSMEHFRWGKPV-amide	1664.80
Proopiomelanocortin	α-MSH, Met-oxidized	Ac-SYS-Mox-EHFRWGKPV-amide	1680.80
Proopiomelanocortin	DiAc-α-MSH	DiAc-SYSMEHFRWGKPV-amide	1706.81
Proopiomelanocortin	DiAc-α-MSH, Met-oxidized	DiAc-SYS-Mox-EHFRWGKPV-amide	1722.81
Proopiomelanocortin	JP	AEEEAVWGDGSPEPSPRE-amide	1940.86
Proopiomelanocortin	Ac-JP	Ac-AEEEAVWGDGSPEPSPRE-amide	1982.87
Proopiomelanocortin	Arg-JP	RAEEEAVWGDGSPEPSPRE-amide	2096.96
Proopiomelanocortin	Glycosylated JP	AEEEAVWGDGSPEP-HexHexNAcS-PRE-amide	2305.99

All masses listed here are monoisotopic. Abbreviations: MSH, melanocyte stimulating hormone; JP, joining peptide.

## Discussion

### Detecting Amidation Reaction Intermediates

We identified JP-Gly, derived from pro-opiomelanocortin (POMC), AVP-Gly, from pro-vasopressin-neurophysin, and OT-Gly, from pro-oxytocin-neurophysin. Other POMC-derived peptides such as α-MSH were observed only in their mature forms with C-terminal amidation, mono- or di-N-terminal acetylation, and/or methionine oxidation. JP, an acidic peptide located between γ-MSH and ACTH in POMC, is a normal end-product of POMC processing and has been used as an indicator of corticotrope function, POMC processing, and amidation [40], [41]. AVP and OT, which regulate plasma osmolaltiy and maternal behavior, respectively, are synthesized in the hypothalamus, transported to the neural lobe of the pituitary, and stored there until released [42], [43].

Several factors may have contributed to our inability to detect additional glycine-extended peptides. First, their intrinsic low levels, especially when compared to the levels of their amidated counterparts, render most glycine-extended peptide ions below the MS detection limit. Second, the α-MSH series of peptides, which end in -Val-NH_2_, are better substrates for PAM than JP (-Glu-NH_2_), OT (-Gly-NH_2_), and AVP (-GlyNH_2_), facilitating more complete α-amidation [44]. Third, many of the undetected partially processed peptides have multiple other post-translational modifications [45]; perhaps the temporal formation of the various modifications determines whether specific partially processed peptides can be detected.

### MS-based Label-free Quantitation

It is worth noting that most quantitative studies focus on the same peptides in multiple samples following different manipulations. Here, on the other hand, we investigated how the relative levels of two related but distinct peptides changed with genetic and dietary manipulations using a label-free LC-MALDI-TOF MS approach. Although the two related peptides have similar amino acid compositions, they may have different ionization efficiencies; therefore, the ratio of partially processed peptides to amidated peptides may not reflect the exact relative amount of the two peptides. However, the ionization efficiency differences for these peptides are eliminated when we compare the ratios of the two related peptides from samples following different manipulations. Therefore, the ratio difference reflects relative peptide level changes in response to various manipulations. This approach should provide a robust comparison of peptides present at very low levels.

### Characterization and Quantification of Hydroxyglycine-extended Peptides

Production of amidated peptides from their glycine-extended precursors involves formation of hydroxyglycine-extended intermediates. This reaction intermediate has been identified in test tube assays and by metabolic labeling [28], but not in tissue, perhaps reflecting its low abundance and intrinsic instability. As early as 1991, Bundgaard et al. [46] reported non-enzymatic, base-catalyzed hydrolysis of hydroxyglycine-extended peptides and their derivatives. Acid- or base-catalyzed deprotonation of the hydroxyl moiety of carbinolamides has also been proposed [47], [48]. Here, we showed that the conversion of hydroxyglycine-extended peptides to their amidated forms also occurs in the gas phase during MS. We employed this conversion as a criterion to aid in our assignment of hydroxyglycine-extended peptides. Although more investigation is needed to reveal the decomposition mechanism, we hypothesize that the acid-catalyzed decomposition of hydroxyglycine-extended peptides is accelerated by an initial decarboxylation step [35], [48].

The ability to detect both JP-Gly and JP-Gly-OH in tissue extracts makes it possible to determine the rate-limiting step in producing JP-NH_2_ by this two-step reaction. Kinetic studies with purified PHM and PAL revealed a higher affinity of PHM for its peptide substrate and a higher turnover number for PAL [49]. The fact that levels of JP-Gly-OH were approximately 74-fold lower than levels of JP-Gly for the same amount of JP-NH_2_ supports the hypothesis that PHM is the rate-limiting enzyme. Assays of purified PAM are consistent with this conclusion; k_cat_ values for PAL are approximately 5-fold higher than k_cat_ values for PHM [49]. Genes encoding bifunctional PAM appear to have evolved from separate genes encoding PHM and PAL [50]. The remarkable sensitivity of hydroxyglycine-extended peptides to their acidic/basic environment may be important in species whose genomes encode monofunctional PHM. Examples include *Drosophila* [4], *Planaria* [51], *Calliactis* [52], and the human parasite *Schistosoma mansoni* [53], where a monofunctional *PHM* gene is not always co-expressed with *PAL*. Compared to WT mice, PAM^+/−^ mice accumulated JP-Gly-OH over amidated JP, suggesting that levels of PAL can become limiting. If the peptidylglycine product of the PHM reaction were passed directly to PAL, one would not expect to see a shift in this ratio. Future work on the stability of hydroxyglycine-extended peptides will provide greater insights into PAL catalytic mechanisms and α-amidation catalyzed by PHM in the absence of PAL.

### PAM Haploinsufficiency and Manipulation of Dietary Copper Produce Small Changes in Glycine-extended Peptides

PAM^+/−^ mice exhibit increased anxiety-like behavior and a decreased ability to regulate body temperature in a cold room [5], [6]. These deficits could reflect abnormal levels of many different amidated peptides or diminished levels of PAM protein in the central nervous system. A γ-secretase-mediated cleavage in the transmembrane domain of PAM releases a soluble cytosolic fragment that accumulates in the nucleus and alters gene expression [7], [8]. Using our ability to quantify peptidylglycine intermediates in the pituitary as a way to predict corresponding changes in the brain, these possibilities can be distinguished.

The LC-MS-based approach used here allowed us to screen peptide content with high throughput and sensitivity. When detectable, we found glycine-extended peptides present at levels 100–1000-fold below those of their amidated products in WT mice. Based on radioimmunoassay data, the TRH-Gly/TRH-NH_2_ ratio in the central nervous system is much higher, perhaps reflecting the diminished affinity of PHM for substrates terminating with a Pro-Gly sequence [5], [12], [13]. Previous radioimmunoassay-based studies of secretin and gastrin, major gastrointestinal peptides, identified high levels of secretin-Gly [54] and gastrin-Gly [14], [15]. In our study, JP-Gly and AVP-Gly were significantly more prevalent in PAM^+/−^ mice than in WT mice, but the glycine-extended peptides were still very minor components.

In addition, in animals on a copper-deficient diet, we observed an accumulation of JP-Gly in WT mice and enhanced accumulation of JP-Gly in PAM^+/−^ mice. Interestingly, copper deficiency led to an accumulation of AVP-Gly in PAM^+/−^ but not WT mice. It is clear that changes in both PAM and copper levels interact and affect different peptides in distinct ways. Our previous study [5], which utilized immunoassays to distinguish TRH-NH_2_ from TRH-Gly in hypothalamic extracts, demonstrated increased levels of TRH-Gly in copper-deficient WT mice, with no effect of copper supplementation on TRH-Gly levels in PAM^+/−^ mice. Similar responses were observed for pituitary JP-Gly and AVP-Gly in this study. The effects of dietary limitations in copper on the amidation reaction vary substantially for different neuropeptides, perhaps reflecting the properties of the prohormone itself or copper metabolism in that particular cell type.

Although PAM haploinsufficiency and manipulation of dietary copper have the predicted effects on JP-Gly and AVP-Gly levels, the amidated versions of these neuropeptides remain much more prevalent. It is difficult to see how changes in the levels of amidated versus glycine-extended peptide could account for the phenotypic changes observed in PAM^+/−^ mice or the ability of dietary copper supplementation to ameliorate these deficits. If our data for pituitary peptides can be applied to the brain, they strongly suggest that the ability of PAM to affect gene expression and copper metabolism plays a key role in determining the phenotype of PAM^+/−^ and copper deficient mice [5], [7].

### PAM Haploinsufficiency Produces Small Changes in Amidated Peptides

Elimination of the PAM gene prevents the formation of amidated peptides and is lethal in both *Drosophila* [4] and mouse [1]. PAM^+/−^ mice, with lower PAM activity than WT mice, survive to adulthood and reproduce [3], but exhibit a variety of deficits [5], [6]. Consistent with these behavior deficits, our results showed that several amidated peptides, including diAc-α-MSH, α-MSH, and JP, undergo significant level changes (*p*<0.05) due to PAM haploinsufficiency. Interestingly, pituitary levels of these peptides appeared to be higher in PAM^+/−^ mice than in WT mice. Since pituitary levels of these peptides reflect their synthesis and their secretion, further studies are needed to determine the underlying cause.

PAM causes gene expression changes by relaying information from secretory granules to the nucleus [7]. The transcripts encoding aquaporin 1 (*Aqp1*) and secretory leukocyte peptidase inhibitor (*Slpi*) were reduced 12-fold and 7-fold in PAM^+/−^ compared to WT mouse pituitary [7]. Aqp1 plays an essential role in secretory granule biogenesis [55], [56]. PAM^+/−^ mice may have an impaired regulated secretory pathway. Reducing levels of Slpi, which inhibits elastase, cathepsin G, trypsin, and chymotrypsin, may contribute to an increase in POMC processing. Pituitary cells overexpressing PAM showed a decrease in POMC processing [57]. In addition, our results provide direct evidence that α-MSH and JP increase in PAM^+/−^ mice. This was demonstrated, albeit indirectly, in two prior studies [5], [7].

Most of the peptides studied did not show significant changes in the PAM^+/−^ mice. Besides diAc-α-MSH, α-MSH, and JP, we also observed other peptides derived from POMC with or without C-terminal amidation. There was a large variation in the relative levels of these peptides among the biological replicates. These peptides are present in a variety of related forms in mouse pituitary. For example, multiple corticotropin-like intermediate lobe peptides (CLIPs) and phosphorylated CLIP have been detected in mouse pituitary [58]. Additional studies on peptide processing and secretion in PAM^+/−^ mice are needed to better correlate peptide processing and peptide levels with PAM activity, and to uncover other roles for PAM in mouse behavior.
